# Endothelial G protein stimulatory α-subunit is a critical regulator of post-ischemic angiogenesis

**DOI:** 10.3389/fcvm.2022.941946

**Published:** 2022-07-25

**Authors:** Lifan He, Hanlin Lu, Jianying Chu, Xiaoteng Qin, Jiangang Gao, Min Chen, Lee S. Weinstein, Jianmin Yang, Qunye Zhang, Cheng Zhang, Wencheng Zhang

**Affiliations:** ^1^The Key Laboratory of Cardiovascular Remodeling and Function Research, Chinese Ministry of Education, Chinese National Health Commission and Chinese Academy of Medical Sciences, The State and Shandong Province Joint Key Laboratory of Translational Cardiovascular Medicine, Department of Cardiology, Qilu Hospital, Cheeloo College of Medicine, Shandong University, Jinan, China; ^2^Cardiovascular Disease Research Center of Shandong First Medical University, Central Hospital Affiliated to Shandong First Medical University, Jinan, China; ^3^Department of Obstetrics and Gynecology, Qilu Hospital, Shandong University, Jinan, China; ^4^School of Life Sciences and Key Laboratory of the Ministry of Education for Experimental Teratology, Shandong University, Jinan, China; ^5^Metabolic Diseases Branch, National Institute of Diabetes, Digestive, and Kidney Diseases, National Institutes of Health, Bethesda, MD, United States

**Keywords:** angiogenesis, AGGF1, cAMP, CREB, Gsα

## Abstract

Post-ischemic angiogenesis is a vital pathophysiological process in diseases such as peripheral arterial disease (PAD), heart ischemia, and diabetic retinopathy. The molecular mechanisms of post-ischemic angiogenesis are complicated and not fully elucidated. The G protein stimulatory alpha subunit (Gsα) is essential for hormone-stimulated cyclic adenosine monophosphate (cAMP) production and is an important regulator for many physiological processes. In the present study, we investigated the role of endothelial Gsα in post-ischemic angiogenesis by generating adult mice with endothelial-specific Gsα deficiency (Gsα^ECKO^). Gsα^ECKO^ mice had impaired blood flow recovery after hind limb ischemic injury, and reduced neovascularization in allograft transplanted tumors. Mechanically, Gsα could regulate the expression of angiogenic factor with G patch and FHA domains 1 (AGGF1) through cAMP/CREB pathway. AGGF1 plays a key role in angiogenesis and regulates endothelial cell proliferation as well as migration. Knockdown of CREB or mutation of the CRE site on the AGGF1 promoter led to reduced AGGF1 promoter activity. In addition, knockdown of AGGF1 reduced the proangiogenic effect of Gsα in endothelial cells, and overexpression of AGGF1 reversed the impaired angiogenesis in Gsα^ECKO^ mice *in vivo*. The finding may prove useful in designing new therapeutic targets for treatments of post-ischemic angiogenesis-related diseases.

## Introduction

Angiogenesis, describes a process of sprouting from pre-existing vessels and subsequent stabilization of these sprouts by mural cells (1) and it plays a critical role in the physiological development of embryogenesis and cardiovascular maturation. Post-ischemic angiogenesis contributes to tissue repair and vascular remodeling in ischemic diseases such as peripheral arterial disease (PAD) and myocardial infarction, which has caused increasing morbidity and mortality worldwide (2, 3). Hence the identification of new molecules or drugs is critical to establishing effective pharmacologic treatment.

The G protein stimulatory α-subunit (Gsα) is encoded by a complex imprinted gene (*GNAS* in human, *Gnas* in mouse) and it couples to hormones and receptors to activate adenylyl cyclase and is required for hormone-stimulated intracellular cyclic adenosine monophosphate (cAMP) generation (4). cAMP interacts with and activates protein kinase A (PKA), which phosphorylates cAMP response element binding protein (CREB) at Ser133 to increase its transcriptional activity (5). Gsα is ubiquitously expressed and has a vital role in many physiological and pathological processes. We have previously reported that Gsα deficiency in smooth muscle cells greatly decreased the contractility of intestinal smooth muscle (6) and exaggerated angiotensin II-induced abdominal aortic aneurysm formation in mice *in vivo* (7). Germline endothelial-specific deletion of Gsα causes severe embryonic vascular defects and embryonical lethality (8). Gsα knockdown decreased the tube number and total tube length of tube-like structure formation in human umbilical vein endothelial cells (HUVECs) (8). However, the role of Gsα in angiogenesis and the underlying mechanism are not well explored.

Post-ischemic angiogenesis is induced by local tissue ischemia or hypoxic damage (9). Many growth factors, including angiogenic factor with G patch and FHA domains 1 (AGGF1), play important roles in physiological and pathological angiogenesis. AGGF1 is the first gene identified in Klipple-Trenaunay syndrome. Aggf1 gene knockout mice resulted in early haploinsufficient embryonic lethality and vascular defects. In addition, as an angiogenic factor, AGGF1 promoted angiogenesis and vascular development by activating PI3K/AKT signaling (10). Meanwhile, AGGF1 could activate autophagy and enhance angiogenesis in coronary artery disease and myocardial infarction (11). Although AGGF1 is well known to regulate angiogenesis, how its expression is regulated remains poorly understood.

Based on the similar phenotypes revealed by germline endothelial-specific deletion of Gsα or AGGF1 mice, we made the hypothesis that Gsα may regulate endothelial angiogenesis through angiogenic factor AGGF1. In the present study, we have generated mice with tamoxifen-induced Cdh5-CreER^T2^ mediated endothelial Gsα deficiency to explore the role of endothelial Gsα in post-ischemic angiogenesis. Our results showed that Gsα deficiency in endothelial cells led to impaired ischemic angiogenesis and Gsα/cAMP/CREB signaling pathway played a critical role in the maintenance of normal angiogenesis through the regulation of angiogenic factor AGGF1 expression.

## Materials and methods

### Generation of endothelial-specific G protein stimulatory α-subunit-knockout mice

All mice were in a C57BL/6J background. The endothelial-specific Gsα knockout (Gsα^ECKO^) mice were generated as follows. Gsα^flox/flox^ mice (12) were bred with Cdh5-CreER^T2^ mice (13) (a gift from Prof. Yulong He in the Soochow University) to obtain Gsα^flox/+^/Cre^+^ mice which were intercrossed to generate Gsα^flox/flox^/Cre^+^ mice. Six-week-old male Gsα^flox/flox^/Cre^+^ mice were intraperitoneally injected with tamoxifen (1 mg/day; Sigma-Aldrich, St. Louis, MO, United States) for 5 consecutive days to generate Gsα^ECKO^ mice. The littermate Gsα^flox/flox^/Cre^–^ mice were treated with the same dose of tamoxifen as the controls (CTR). Genotyping involved using genomic DNA from mouse tails and PCR was performed with the following primers: Cdh5-Cre forward: ACTAAACTGGTCGAGCGATGGA; reverse: TGTCCAGACCAGGCCAGGTA; Gsα-flox forward: GCTCTCCCCCTCTTTCTCTC; reverse: GCAGGATCCTCA TCTGCTTC. All animal procedures were approved by and conducted in accordance with the National Institutes of Health Guidelines and with the approval of the Animal Care and Use Committee of Shandong University (Approval No. DWLL-2018-018).

### Hind limb ischemia model

CTR and Gsα^ECKO^ mice at 8 weeks old underwent hind limb ischemia as described (14). Mice were anesthetized with ketamine and xylazine (100 mg/kg + 5 mg/kg, respectively, i.p.) and adequate anesthesia was confirmed by the absence of the pedal reflex. The hair of mice from the low abdomen to the toe was removed. The skin was cut about 7 mm below the inguinal region and 3 mm above it. Then fat tissue was removed and the neurovascular bundle was explored. The femoral artery was separated and ligated with a 10–0 silk suture. The first ligation site was below the inguinal ligament and the second site was proximal to the saphenous artery. Blood flow was measured before, on 1 and 3 days, 1 and 2 weeks after femoral artery ligation by using Laser Doppler and PeriScan PIM 3 System (Perimed AB, Jarfalla, Sweden). The CTR and Gsα^ECKO^ mice were injected with adenovirus expressing LacZ or AGGF1 (10^9^ PFU/mouse GeneChem, Shanghai, China) through tail vein 1 day prior to surgery, and other procedures were the same as above. Two weeks after femoral artery ligation, the mice were euthanized using ketamine and xylazine (500 mg/kg + 25 mg/kg, respectively, i.p.) followed by exsanguination, and gastrocnemius muscle tissue was removed. Data were expressed as the ratio of ischemic to non-ischemic hindlimb blood flow for each animal at each time point.

### *In vivo* angiogenesis assay with matrigel plug

Eight-week-old male CTR and Gsα^ECKO^ mice were anesthetized as described above and injected subcutaneously into the flank with 300 μL growth factor-reduced Matrigel (Corning, NY, United States), mixed with vascular endothelial growth factor (VEGF-100 ng/mL; PeproTech, Cranbury, NJ, United States) and heparin sodium (40 U/mL; Bio Vision, Milpitas, CA, United States). After 7 days, mice were euthanized as described above and matrigel plugs were removed. Implants were then embedded with O.C.T compound for CD31 staining.

### Tumor angiogenesis assay

Eight -week old male CTR and Gsα^ECKO^ mice were anesthetized as described above and hair in the flank region was removed. A total of 1 million Lewis lung carcinoma (LLC) cells mixed with matrigel were injected subcutaneously into the dorsal region of each mouse. Mice were sacrificed 14 days after the injection, and tumors were collected for imaging, measurement of the volume (length × height × width × 0.5236), and immunofluorescence assay with CD31.

### Spheroid-based *in vitro* angiogenesis assay

HUVECs were detached from cell culture plates and resuspended in endothelial cell medium. 4 mL of endothelial cell medium containing 12 × 10^4^ cells was mixed carefully with 1 mL of methocel stock solution (1.2% methylcellulose; Sigma-Aldrich). Next, 25 μL of the cell solution mixture was placed onto the lid of a cell culture dish which was then inverted. The bottom was filled with PBS and the culture dish was incubated in a humidified cell culture incubator for 24 h, and the spheroid shape formed. The hanging drops were then washed off by PBS. The cell spheroids were resuspended with 0.5 mL of methocel solution containing 20% FBS. Afterward, rat collagen I (2 mg/mL; Salarbio, Beijing, China) was added and avoid bubbles. The spheroid-collagen solution was added to a 24-well plate which had been pre-warmed. Sprouts were photographed under Nikon inverted microscope.

### Cell culture

HUVECs were purchased from ScienCell (Santiago, MN, United States, Lot Number 28433), and cultured with endothelial cell medium (ScienCell). The fourth to sixth generations of cells were used for experiments. At 80∼90% confluence, HUVECs were transfected with control or Gsα siRNA (GenePharma, Shanghai) using Lipofectamine RNAiMAX transfection reagent (Thermo Fisher Scientific, Waltham, MA, United States) or infected with adenovirus-expressing GFP or Gsα (Vigenebio, Jinan, China). H89 and forskolin were purchased from Abcam (Cambridge, United Kingdom).

### Western blot analysis

Tissue or cellular proteins were extracted with RIPA lysis buffer containing protease and phosphatase inhibitors. Lysates were separated by SDS-PAGE and transferred to PVDF membranes, which were blotted with specific antibodies: anti-Gsα (Santa Cruz, Dallas, TX, United States), anti-CREB (Cell Signaling Technology, Boston, MA, United States), anti-phospho-CREB Ser133 (Cell Signaling Technology), anti-AGGF1 (Abcam), anti-GAPDH (Proteintech, Chicago, IL, United States), and anti-CyclinD1 (Cell Signaling Technology). Membranes were washed, incubated with secondary antibody, and developed with Immobilon ECL Ultra Western HRP Substrate (Millipore, MA, United States) using Bio-Rad ChemDoc MP system (Bio-Rad, Hercules, CA, United States). The bands were quantified by Image J.

### Immunofluorescence assay

The immunofluorescence assay was as described (15). Briefly, frozen sections were prepared by rinsing with PBS three times. Formalin-fixed paraffin embedded slides underwent heat-induced epitope retrieval with sodium citrate buffer and were then blocked with 10% goat serum in PBS for 1 h. Immunofluorescence assay involved anti-CD31 (Abcam) and anti-Gsα (Santa Cruz) primary antibodies followed by goat polyclonal secondary antibody to mouse IgG-H&L and goat polyclonal secondary antibody to rabbit IgG-H&L (Alexa Fluor 594) (both Abcam). Sections were covered with mounting medium with DAPI (Abcam) and covered. Images were taken under a fluorescence microscope (Nikon-U).

### RNA extraction and quantitative RT-PCR

The total RNA was extracted from HUVECs by using the RNAfast200 kit (QIAGEN, Dusseldorf, Germany). The procedures were performed following the manufacturer’s instructions. cDNA was generated by using the PrimeScript RT reagent kit with gDNA Eraser (Takara, Otsu, Shiga Prefecture, Japan). PCR involved using TB Green Premix Ex Taq II (Takara) with the Roche LightCycler 480II and the following primer sequences. AGGF1: forward 5′-GGAGGA ATGAAAACGCCGATCC-3′ and reverse 5′-AAACCGCTCT CGTGCTTTGT-3′. β-actin: forward 5′-CATGTACGTTGCT ATCCAGGC-3′ and reverse 5′-CTCCTTAATGTCACGCA CGAT-3′.

### Cyclic adenosine monophosphate (cAMP) assay

cAMP assay of HUVECs or lung tissue was performed using the cAMP ELISA kit (Enzo Life Sciences, New York, NY, United States). The procedures were performed following the manufacturer’s instructions.

### Chromatin immunoprecipitation assay

ChIP assay of HUVECs was performed using the Simple ChIP Plus Enzymatic Chromatin IP Kit (Cell Signaling Technology). Briefly, HUVECs were cross-linked with formaldehyde. DNA was treated by sonication and incubated with 1 μg rabbit IgG or anti-CREB antibody (Cell Signaling Technology). Immunoprecipitation was performed with the magnetic beads, and 2 μL immunoprecipitated DNA underwent PCR with the following primers: 5′-CTCTCCACGCCCTCAGGTAA-3′ and 5′-CGTCGGATAAGCAGTCGGAA-3′.

### Transcriptome sequencing

Total RNA was extracted from GFP and Gsα virus transfected HUVECs. The transcriptome sequencing experiment was performed by Annoroad Gene Technology Company (Beijing, China). The transcriptome library for sequencing was assayed using Agilent 2100 RNA Nano 6000 Assay (Agilent Technologies, CA, United States) following the manufacturer’s recommendations. The libraries were sequenced on the Illumina platform using the PE150 module. The differentially expressed genes were identified with *P*-value < 0.05 and a fold-change of > 1.5 between the two groups. The sequencing data has been deposited in Gene Expression Omnibus (GEO).

### Luciferase reporter assay

The DNA fragment from the human AGGF1 promoter was cloned into pGL3 Basic (Promega, Madison, WI, United States) to generate the wild-type Luc construct. The mutant construct with deletion of the CRE site in the AGGF1 promoter was generated by using the Quick Change II Site-Directed Mutagenesis Kit (Stratagene, La Jolla, CA, United States). For luciferase assay, the luciferase reporter plasmid was transfected into HEK-293T cells in 24-well plates by using lipofectamine 2000 reagent (Thermo Fisher Scientific). The p-RL-TK plasmid carrying the Renilla luciferase under the control of the thymidine kinase promoter was co-transfected as internal control for transfection efficiency. After 24 h, cells were infected with the adenovirus expressing GFP or Gsα and luciferase activity was analyzed by using the dual luciferase assay kit (Beyotime, Nantong, China).

### Wound healing assay

HUVECs were seeded in 6-well plates transfected with control or AGGF1 siRNA for 24 h and infected with the adenovirus expressing GFP or Gsα virus. Then endothelial monolayers were scratched with a 200 μL tip and wound closures were analyzed at 48 h after scratching.

### Statistical analysis

Data are expressed as mean ± SEM and were analyzed by using GraphPad Prism 7 (GraphPad Software Inc., San Diego, CA). Statistical comparisons of two groups involved Student’s *t*-test and more than three groups involved by One-Way ANOVA and Bonferroni post-tests. *P* < 0.05 was considered statistically significant.

## Results

### Generation of endothelia-specific G protein stimulatory α-subunit deficient mice

**FIGURE 1:**
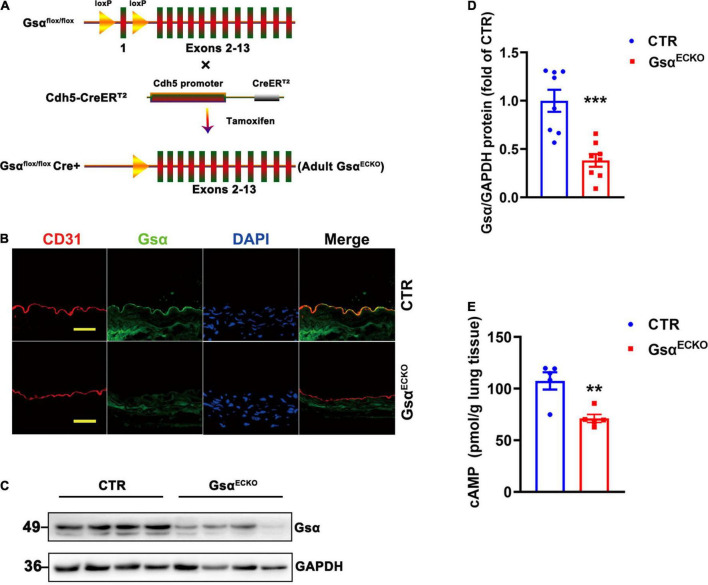
Generation of endothelia specific-Gsα knockout mice. **(A)** Schematic diagram of generation of endothelial-specific Gsα knockout mice. **(B)** Representative immunofluorescence staining for Gsα, CD31 for endothelial cells, and DAPI for nuclei in aortas from control (CTR) and Gsα^ECKO^ mice. Scale bar, 100 μm. **(C,D)** Western blot analysis and quantification of Gsα protein in lung tissues of CTR and Gsα^ECKO^ mice. *n* = 8/group. **(E)** The cAMP level was measured in lung tissue of CTR and Gsα^ECKO^ mice. *n* = 5/group. **P < 0.01, ***P < 0.001 vs. CTR (unpaired Student’s *t*-test). Data are mean ± SEM.

Gsα^flox/flox^ mice were cross-bred with Cdh5-CreER^T2^ mice to generate Gsα^flox/+^/Cre^+^ mice, and the offspring were further intercrossed to obtain Gsα^flox/flox^/Cre^+^ mice. To avoid the impact of loss of Gsα in endothelial cells on the development of mice, we induced Cdh5-CreER^T2^ activity when mice were 6 weeks old by intraperitoneal injection of tamoxifen for 5 consecutive days to delete Gsα in endothelial cells (referred to as Gsα^ECKO^ mice; Figure 1A). Littermate Gsα^flox/flox^/Cre^–^ mice were used as controls (CTR). To confirm Gsα deficiency in endothelial cells of Gsα^ECKO^ mice, an immunofluorescence assay was used to detect Gsα protein expression in aortas endothelium from CTR and Gsα^ECKO^ mice with endothelial cells were labeled by CD31staining. Gsα protein was expressed in the endothelium of aortas from CTR but not in the endothelium from Gsα^ECKO^ mice (Figure 1B). The western blotting analysis also showed that Gsα protein level significantly decreased in the lung tissue of Gsα^ECKO^ mice as compared with that of CTR mice (Figures 1C,D), which was consistent with decreased cAMP level in the lung tissue of Gsα^ECKO^ mice (Figure 1E). Thus, Gsα was effectively deleted in endothelial cells of Gsα^ECKO^ mice.

### Endothelial G protein stimulatory α-subunit deficiency impairs blood flow recovery after ischemic injury

**FIGURE 2:**
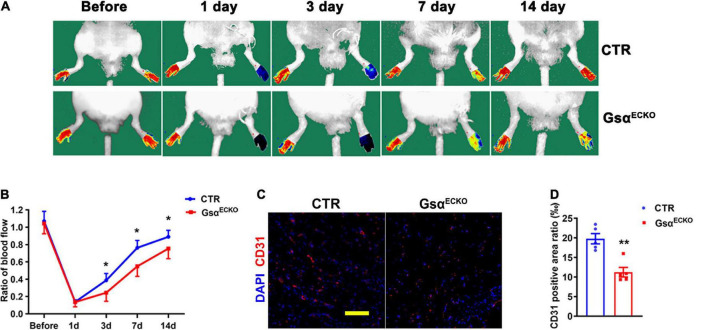
Endothelial Gsα deficiency impairs blood flow recovery after ischemic injury. **(A)** Representative images of laser doppler blood flow before or on days 1, 3, 7, and 14 after femoral artery resection. **(B)** Blood flow in ischemic hind limbs. Data are expressed as a ratio of ischemic to non-ischemic limb perfusion. *n* = 6/group. **(C)** Representative immunofluorescence staining at day 14 of CD31 for endothelial cells and DAPI for nuclei in gastrocnemius muscle from ischemic hind limbs of CTR and Gsα^ECKO^ mice. Scale bar, 100 μm. **(D)** Quantification of CD31-positive area in **(C)**, *n* = 5/group, *P < 0.05, **P < 0.01 vs. CTR (unpaired Student’s *t*-test). Data are mean ± SEM.

To reveal the role of endothelial Gsα in post-ischemic angiogenesis *in vivo*, CTR and Gsα^ECKO^ mice were subjected to hindlimb ischemia by ligation of the left femoral artery. On laser doppler perfusion imaging of distal ischemia foot or normal foot, blood perfusion of distal limb was significantly impaired in Gsα^ECKO^ mice at days 3, 7, or 14 after ischemic injury as compared with CTR mice (Figures 2A,B), which showed that endothelial Gsα deficiency impaired the post-ischemia angiogenesis. Mice were sacrificed at day 14 post-ligation of femoral artery and the gastrocnemius muscle was harvested for immunofluorescence assay to analyze capillary density, which was labeled with CD31 antibody. Consistent with the results of blood perfusion, the capillary density in skeletal muscle on day 14 after ischemic injury was significantly decreased in Gsα^ECKO^ mice as compared with CTR (Figures 2C,D). Collectively, our data indicated that endothelial Gsα plays a critical role in post-ischemic angiogenesis.

### Endothelial G protein stimulatory α-subunit deficiency inhibits neovascularization and tumor angiogenesis *in vivo*

**FIGURE 3:**
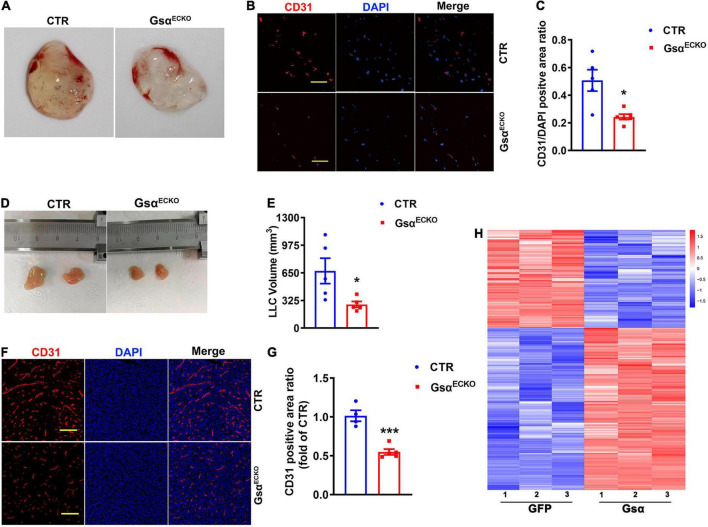
Loss of endothelial Gsα inhibits neovascularization and tumor growth *in vivo*. **(A)** Representative matrigel plugs retrieved from CTR and Gsα^ECKO^ mice. **(B)** Representative immunofluorescence staining of CD31 and DAPI in matrigel plugs. Scale bar, 50 μm. **(C)** Quantification of the ratio of CD31-positive to DAPI staining. *n* = 5 for CTR; *n* = 6 for Gsα^ECKO^. **(D)** Images of LLCs harvested from CTR and Gsα^ECKO^ mice. **(E)** Quantification of tumor volume. *n* = 5/group. **(F)** Representative immunofluorescence staining of CD31 and DAPI from LLC tumors. Scale bar, 100 μm. **(G)** Quantification of the ratio of CD31-positive area. *n* = 4 for CTR, *n* = 5 for Gsα^ECKO^, *P < 0.05, ***P < 0.001 vs. CTR (unpaired Student’s *t*-test). Data are mean ± SEM. **(H)** Transcriptome sequencing of genes with > 1.5- fold upregulation or > 1.5-fold down-regulation in Gsα virus transfected HUVEC compared with GFP.

We next studied neovascularization using matrigel plug assay which had been widely used to assess angiogenesis *in vivo* and was a model of neovascularization occurring with ischemia and inflammation. The matrigel hardened into a plug, which was invaded by immune cells and becomes vascularized (16). Matrigel was injected into a subcutaneous location of CTR and Gsα^ECKO^ mice and removed at 7 days. We observed that the plug from Gsα^ECKO^ mice displayed a less degree of yellowish color compared with that from CTR (Figure 3A), indicating that the initial angiogenesis activity was lower in Gsα^ECKO^ mice. An immunofluorescence assay was used to evaluate blood vessel formation and perfusion within the plugs. As expected, there was less CD31 staining in Matrigel plugs from Gsα^ECKO^ than in CTR mice (Figures 3B,C). In addition, CTR and Gsα^ECKO^ mice underwent allograft transplantation of LLC, and tumor growth was monitored for 14 days. LLC tumors grew slower in Gsα^ECKO^ than CTR mice (Figures 3D,E). Consistently, the vasculature with CD31 staining was less dense in LLC tumors of Gsα^ECKO^ than CTR mice (Figures 3F,G), suggesting that delayed LLC tumor growth may result from poor angiogenesis in Gsα^ECKO^ mice. These data indicated that endothelial Gsα promoted inflammatory neovascularization and tumor angiogenesis in mice, which was consistent with the results that loss of Gsα in endothelial cells impaired post-ischemic angiogenesis. To investigate the specific mechanism of Gsα functioning in endothelial cell, a transcriptome sequencing assay of HUVECs infected with GFP or Gsα virus were conducted to profile differentially expressed genes. As shown in the heatmap (Figure 3H), we found 307 genes with > 1.5-fold upregulation and 184 genes with 1.5-fold down-regulation in Gsα virus transfected HUVEC compared with GFP. We further determined the expression of specific genes by both knockdown and overexpression strategies.

### G protein stimulatory α-subunit deficiency leads to decreased angiogenic factor with G patch and FHA domains 1 expression in endothelial cells

**FIGURE 4:**
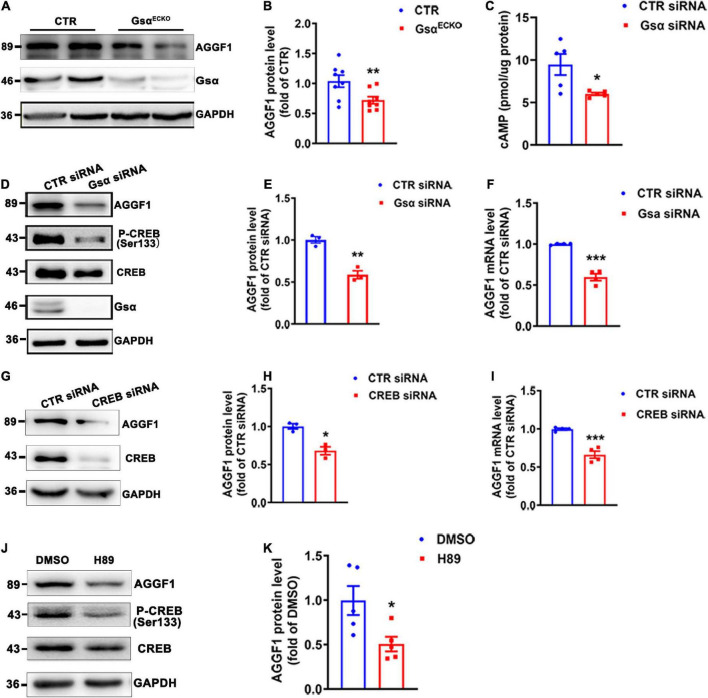
Gsα deficiency decreases AGGF1 expression. **(A,B)** Western blot analysis and quantification of AGGF1 protein levels lung tissues from CTR and Gsα^ECKO^ mice. *n* = 8/group. **(C)** cAMP level was measured in HUVECs transfected with CTR siRNA or Gsα siRNA. *n* = 5/group. **(D,E)** Western blot analysis and quantification of AGGF1 in HUVECs transfected with CTR siRNA or Gsα siRNA. *n* = 3/group. **(F)** RT-qPCR analysis of AGGF1 mRNA level in HUVECs transfected with CTR siRNA or Gsα siRNA. *n* = 4/group. **(G,H)** Western blot analysis and quantification of AGGF1 in HUVECs transfected with CTR or CREB siRNA. *n* = 3/group. **(I)** RT**-**qPCR analysis of AGGF1 mRNA level in HUVECs transfected with CTR or CREB siRNA. *n* = 4/group. **(J,K)** Western blot analysis and quantification of AGGF1 in HUVECs treated with DMSO or H89 (10 μM) for 24 h. *n* = 5/group, *P < 0.05, **P < 0.01, ***P < 0.001 vs. DMSO (unpaired Student’s *t*-test). Data are mean ± SEM.

AGGF1 has been identified as a factor essential for both physical angiogenesis and pathological tumor angiogenesis *in vivo* (17). We therefore detected whether Gsα could regulate AGGF1 expression and observed that AGGF1 protein levels were markedly decreased in lung tissue and isolated lung endothelial cells of Gsα^ECKO^ mice as compared with CTR (Figures 4A,B and Supplementary Figure 1A). The immunofluorescence staining of AGGF1 from ischemic gastrocnemius muscle of CTR and Gsα^ECKO^ mice showed decreased AGGF1 expression in Gsα-deficient endothelial cells (Supplementary Figure 1B). Similar to the results obtained from *in vivo* study (Figures 4A,B), Gsα knockdown with siRNA reduced the protein and mRNA levels of AGGF1 in HUVECs (Figures 4D–F). Since Gsα is required for receptor-stimulated cAMP generation and subsequent CREB activation, we thus examined whether Gsα/cAMP/CREB signaling was responsible for the decreased AGGF1 expression in Gsα or CREB-deficient endothelial cells. As expected, knockdown of Gsα with siRNA decreased cAMP levels and phosphorylation of CREB in HUVECs (Figures 4C,D). Also, CREB knockdown with siRNA suppressed the protein and mRNA levels of AGGF1 (Figures 4G–I). Moreover, H89, as a PKA inhibitor, could inhibit CREB phosphorylation and AGGF1 expression in HUVECs (Figures 4J,K). Thus, Gsα deficiency decreased AGGF1 expression in endothelial cells.

### G protein stimulatory α-subunit regulates angiogenic factor with G patch and FHA domains 1 expression *via* cAMP response element binding protein-binding to the angiogenic factor with G patch and FHA domains 1 promoter

**FIGURE 5:**
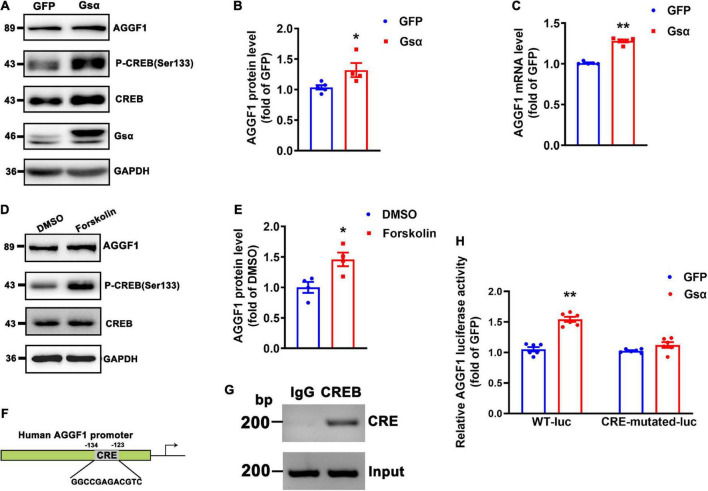
Gsα regulates AGGF1 expression *via* CREB in endothelial cells. **(A,B)** Western blot analysis and quantification of AGGF1 in HUVECs infected with adenovirus-expressing GFP or Gsα. *n* = 5 for GFP; *n* = 4 for Gsα, *P < 0.05 vs. GFP (unpaired Student’s *t*-test). **(C)** RT-qPCR analysis of AGGF1 mRNA in HUVECs infected with adenovirus-expressing GFP or Gsα. *n* = 5/group, **P < 0.01 vs. GFP (unpaired Student’s *t*-test). **(D,E)** Western blot analysis and quantification of AGGF1 in HUVECs treated with forskolin (10 μM) for 24 h. *n* = 4/group, *P < 0.05 vs. DMSO (unpaired Student’s *t*-test). **(F)** Predicted CRE site in the human AGGF1 promoter. **(G)** Binding of CREB to the CRE of the AGGF1 promoter was shown by Chromosome immunoprecipitation assay. **(H)** Luciferase activity in HEK-293T cells transfected with wild-type or a mutant AGGF1 promoter-luciferase construct and infected with adenovirus-expressing GFP or Gsα for 24 h. Results of luciferase promoter assay were firefly/Renilla luciferase activity, *n* = 6/group, **P < 0.01 vs. GFP (One-Way ANOVA and Bonferroni post-tests). Data are mean ± SEM.

Gsα overexpression increased CREB Ser133 phosphorylation and AGGF1 protein and mRNA levels (Figures 5A–C). Forskolin (cAMP activator) treatment induced CREB activity, and increased AGGF1 expression (Figures 5D,E). To determine whether Gsα regulated AGGF1 expression *via* CREB-mediated transcription, the promoter of AGGF1 was analyzed by searching the Transcription Factor Database,^1^ an Internet-based transcription-factor binding-site program, one CRE site in the AGGF1 promoter was identified (Figure 5F). To test whether CREB bound to the deductive CRE on the AGGF1 promoter and regulated its expression, we performed chromatin immunoprecipitation assay. The result demonstrated that CREB could bind to the CRE site in the AGGF1 promoter (Figure 5G). To further analyze the role of the CREB binding site in AGGF1 promoter activity, we deleted the core CREB binding site in CRE, inserted it into a luciferase plasmid, and tested it in HEK-293T cells. Gsα overexpression significantly increased luciferase activity from the wild-type but not CRE-mutant AGGF1 promoter (Figure 5H). The results indicated that the CREB binding site of the AGGF1 promoter was required for Gsα-induced AGGF1 gene expression. Thus, our data confirmed that AGGF1 gene expression was stimulated by Gsα/cAMP/CREB signaling pathway in endothelial cells.

### Knockdown of angiogenic factor with G patch and FHA domains 1 attenuates the proangiogenic effect of G protein stimulatory α-subunit in endothelial cells

**FIGURE 6:**
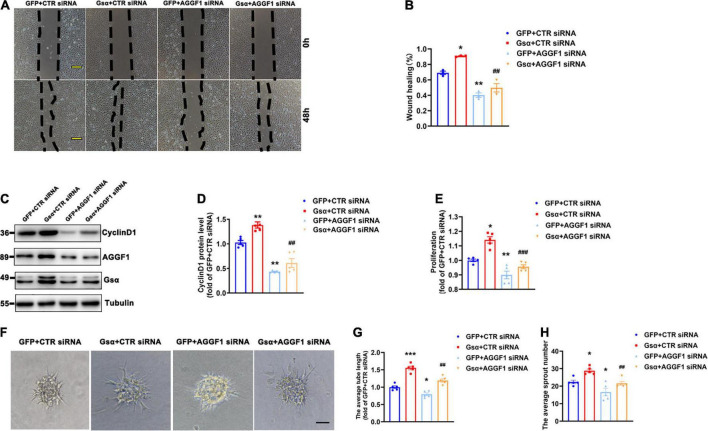
Knockdown of AGGF1 attenuates the proangiogenic effect of Gsα in endothelial cells. **(A)** Representative scratch wound assays of HUVECs transfected with CTR or AGGF1 siRNA and infected with GFP or Gsα virus. Scale bar, 100 μm. **(B)** Quantification of scratch wound closure of HUVECs shown in **(A)**. *n* = 3/group. **(C,D)** Western blot analysis and quantification of CyclinD1 protein level in HUVECs. *n* = 5/group. **(E)** Cell proliferation was measured in HUVECs by using a CCK8 ELISA kit. *n* = 5/group. **(F)** The spheroid assay of HUVECs transfected with CTR or AGGF1 siRNA and infected with GFP or Gsα virus. Scale bar, 100 μm. **(G,H)** Quantification of the average sprout number and tube length of HUVECs transfected with CTR or AGGF1 siRNA and infected with GFP or Gsα virus. *n* = 5/group. *P < 0.05, **P < 0.01, ***P < 0.001 vs. GFP + CTR siRNA, ^##^*P* < 0.01, ^###^*P* < 0.001 vs. Gsα + CTR siRNA (One-Way ANOVA and Bonferroni post-tests). Data are mean ± SEM.

To investigate whether endothelial Gsα contributes to angiogenesis *via* AGGF1, we introduced CTR or AGGF1 siRNA into HUVECs and infected these cells with adenovirus expressing GFP or Gsα. The result of the wound healing assay showed that Gsα promoted wound healing by promoting endothelial cell migration, which was attenuated by AGGF1 knockdown (Figures 6A,B). In addition, Gsα overexpression increased Cyclin D1 protein levels and cell proliferation ability, both these Gsα-induced incidents were significantly inhibited by knockdown of AGGF1 (Figures 6C–E). The spheroid assay indicated that Gsα overexpression enhanced the endothelial sprout numbers and tube length, which were diminished by knockdown of AGGF1 (Figures 6F–H). Thus, the results demonstrate that endothelial Gsα could regulate angiogenesis at least partly through AGGF1.

### Overexpression of angiogenic factor with G patch and FHA domains 1 alleviates the impaired angiogenesis in Gsα^ECKO^ mice

**FIGURE 7:**
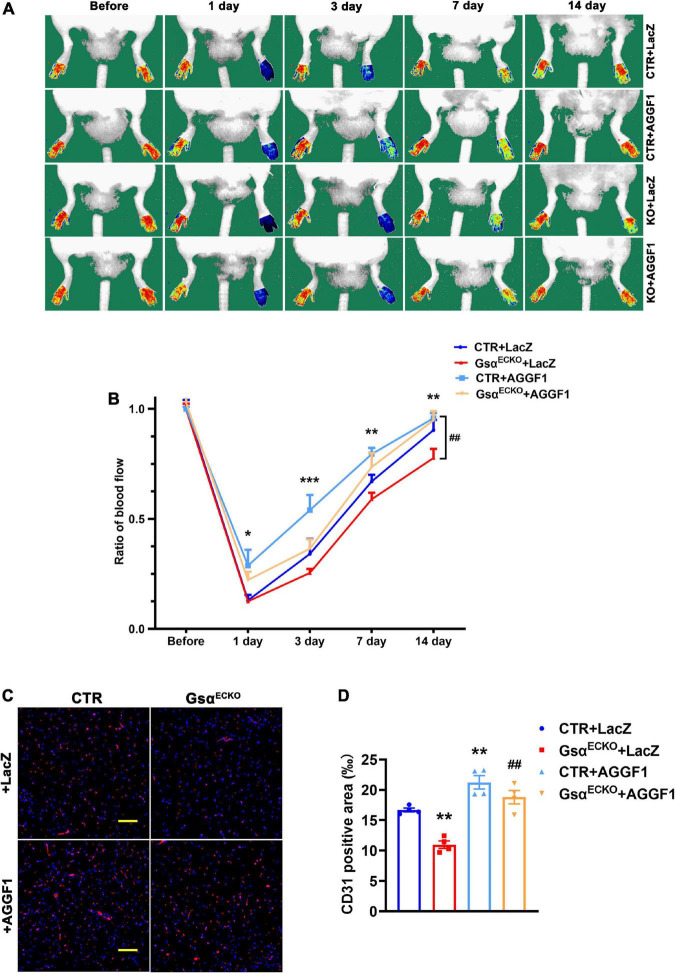
Adenoviral overexpression of AGGF1 alleviates the impaired angiogenesis in Gsα^ECKO^ mice. **(A)** CTR and Gsα^ECKO^ mice were injected with adenovirus expressing LacZ or AGGF1 (10^9^ PFU/mouse) followed by hind-limb ischemia assay. Representative images of blood flow before or on days 1, 3, 7, and 14 after femoral artery ligation. **(B)** Quantification of blood flow in ischemic hind limbs. Data are expressed as a ratio of ischemic to non-ischemic hind-limb perfusion. *n* = 5/group, *P < 0.05, **P < 0.01, ***P < 0.001 vs. CTR + LacZ; ^##^*P* < 0.01 vs. Gsα^ECKO^ + LacZ (One-Way ANOVA and Bonferroni post-tests). **(C)** Representative immunofluorescence staining of CD31 and DAPI in ischemic gastrocnemius muscle of mice. Scale bar, 100 μm. **(D)** Quantification of CD31-positive area in **(C)**. *n* = 4/group, **P < 0.01 vs. CTR + LacZ; ^##^*P* < 0.01 vs. Gsα^ECKO^ + LacZ (One-Way ANOVA and Bonferroni post-tests). Data are mean ± SEM.

To verify whether Gsα regulated ischemic angiogenesis *via* AGGF1 *in vivo*, we administrated adenovirus expressing LacZ or AGGF1 to CTR and Gsα^ ECKO^ mice *via* tail vein injection, then ligated the left femoral artery to induce hindlimb ischemia in these mice. Immunofluorescence staining was performed to validate the overexpression of AGGF1 adenovirus in endothelial cells of mice gastrocnemius muscle (Supplementary Figure 1C). As shown in Figures 7A,B, the degree of ischemic severity was significantly less in CTR mice treated with AGGF1 compared with their counterparts treated with LacZ from day 1 to day 14 post-ligation surgery, confirming that AGGF1 enhanced blood flow recovery after ischemic injury. Consistent with the result shown in Figure 2B, Gsα^ECKO^ mice had delayed blood flow recovery in the ischemic hindlimb, but the treatment of AGGF1 significantly ameliorated ischemia with improved blood flow recovery compared to Gsα^ECKO^ mice treated with LacZ (Figures 7A,B). Similarly, at 14 days after ischemic injury, Gsα^ECKO^ mice had low capillary density evidenced by CD31 staining compared to that of CTR, and treatment of AGGF1 significantly increased the capillary density in skeletal muscle of ischemic hindlimb from both CTR and Gsα^ECKO^ mice (Figures 7C,D). Our data indicates that AGGF1 plays an important role in Gsα-mediated regulation after ischemic injury.

## Discussion

In this study, we investigated the effect of endothelial-specific Gsα deficiency on post-ischemic angiogenesis using the adopted hind limb ischemia model, and showed that Gsα deficiency in endothelial cells impaired post-ischemic angiogenesis leading to decreased blood flow recovery of ischemia limb. Meanwhile, *in vivo* matrigel migration and tumor angiogenesis assays showed fewer newly formed vessels and smaller tumor sizes in Gsα^ECKO^ mice than in CTR. Mechanically, Gsα deficiency reduced the transcriptional activity of CREB and subsequently attenuated the expression of pro-angiogenic AGGF1. In contrast, overexpression of AGGF1 alleviated the impaired angiogenesis caused by endothelial Gsα deletion. Our study provides evidence that endothelial Gsα plays a vital role in post-ischemic angiogenesis through the regulation of AGGF1.

The importance of the ubiquitously expressed Gsα in the maintenance of normal functions in divergent organs had been demonstrated in many studies using tissue-specific Gsα transgenic and knockout mouse models (18–21). The indispensable role of Gsα in the control of vascular development was indicated by the fact that germline endothelia-specific Gsα deficiency led to embryonic lethality due to massive hemorrhage and a disorganized vasculature (8). Laminar and disturbed flow could activate endothelial CALCRL/Gs-mediated signaling and result in the inhibition of the NF-κB activation (22). Shear stress through PIEZO1 induced the release of adrenomedullin which activated its Gs-coupled receptor and increased cAMP levels to promote the phosphorylation of endothelial NO synthase (eNOS) at serine 633 through PKA, leading to the activation of the enzyme (23). In the present study, we generated a mouse model with tamoxifen-induced endothelia-specific Gsα knockout, as well as silenced Gsα in HUVECs and studied the consequences of Gsα deficiency in endothelial cells *in vivo* and *in vitro*. Our results showed that Gsα enhanced endothelial angiogenesis through stimulation of angiogenic factor AGGF1.

The Gsα/cAMP signaling primarily activates PKA, a serine/threonine protein kinase, which phosphorylates enzymes and other cellular substrates to regulate many physiologic processes. PKA can stimulate gene expression *via* phosphorylation of transcription factors such as CREB. Alternatively, Gsα/cAMP signaling mediates some of its actions by stimulating cAMP-regulated guanine nucleotide exchange factors leading to the activation of Ras-like proteins such as Rap1 (24), and Gsα/cAMP signaling may also mediate its actions by stimulating other downstream effectors, such as Ca^2+^ channels (25). Some researchers showed that ETAR (Endothelin-A receptor)-mediated Gαs activation, stimulated AC/cAMP/PKA signaling, which had been shown to limit tumor growth in numerous carcinoma-derived cell lines (26, 27). Other research displayed that EP4 -to-PKA

increased the angiogenic response (28). In our study, we showed that endothelial Gsα deficiency destroyed post-ischemic angiogenesis in ligating limb of mice, as well as tumor angiogenesis. In contrast, overexpressing Gsα induced CREB phosphorylation and elevated AGGF1 mRNA and protein levels in HUVECs, which were in concert with enhanced AGGF1 promoter activity. Meanwhile, given the complexity of Gsα and its downstream pathways, we believe endothelial Gsα may regulate angiogenesis through other pathways besides AGGF1.

Multiple intracellular pathways may affect angiogenesis. Under control of the VEGF-VEGFR and Delta-Notch signaling pathways, tip cells cooperating with stalk cells form a nascent vascular lumen (29). The Ang/Tie2 pathway is essential for the sprouting and branching of vessels in angiogenesis (10). Recently, endothelial cell metabolism has been identified as a driver rather than a bystander effect of angiogenesis in health and disease (30). For example, PFKFB3, CPT1a, and AIBP not only participate in endothelial cell metabolism but also affect angiogenesis (30). AGGF1, as an angiogenic factor, acts on endothelial cells in an autocrine fashion (31). Endothelial AGGF1 promotes angiogenesis and vascular development by activating PI3K/AKT; and is required for maintaining vascular integrity by regulating the phosphorylation and membrane localization of VE-cadherin (10). In addition, AGGF1 can induce autophagy by activating JNK during angiogenesis (11). Although GATA1 and P65 have been identified as transcriptional factors of AGGF1 (32, 33), the mechanism responsible for the regulation of AGGF1 gene expression has not been fully clarified. Our study reveal that Gsα/cAMP/CREB signaling stimulates AGGF1 gene expression.

Therapeutic angiogenesis has been shown to revascularize ischemic heart tissue, reduce the progression of tissue infarction, and evade the need for invasive surgical procedures or tissue/organ transplantations (34). Our study strengthens the understanding of the function of Gsα in the angiogenic process and indicates a possibility of promoting Gsα/cAMP signaling for the treatment of ischemia-related diseases. Nevertheless, findings from this study are not only confined to cardiovascular diseases. The results of employment of the allograft transplantation of LLC onto CTR and Gsα^ECKO^ mice showed that Gsα deficiency in endothelial cells could inhibit tumor growth, which may credit to insufficient neovascularization and nutrient supply to the tumor. Thus, our results suggest that activation and inactivation of Gsα/cAMP signaling may have opposite benefits for ischemia-related disease therapy and cancer therapy, respectively. Overall, our results provide evidence indicating the critical role of endothelial Gsα in angiogenesis and reveal the mechanism underlying the regulation of AGGF1, which may provide a new strategy for the treatment of ischemia-related diseases.

## Data availability statement

The original contributions presented in this study are publicly available. This data can be found here: GEO database, accession GSE206934 (private accession).

## Ethics statement

The animal study was reviewed and approved by the Animal Care and Use Committee of Shandong University. Written informed consent was obtained from the owners for the participation of their animals in this study.

## Author contributions

LH, WZ, and CZ designed the study. LH, HL, JC, and XQ performed the experiments and analyzed the results. LH and WZ drafted the manuscript. JG, MC, LW, JY, QZ, and CZ revised the manuscript for important intellectual contents. All authors read and approved the final manuscript.

## Conflict of interest

The authors declare that the research was conducted in the absence of any commercial or financial relationships that could be construed as a potential conflict of interest.

## Publisher’s note

All claims expressed in this article are solely those of the authors and do not necessarily represent those of their affiliated organizations, or those of the publisher, the editors and the reviewers. Any product that may be evaluated in this article, or claim that may be made by its manufacturer, is not guaranteed or endorsed by the publisher.

## Footnotes

1
http://jaspar.genereg.net

## Supplementary material

Supplementary Figure 1 Deletion of Gsα in endothelial cells reduced the expression of AGGF1. **(A)** Western blot of AGGF1 in isolated lung endothelial cells from CTR and Gsα^ECKO^ mice. **(B)** Immunofluorescent staining of CD31 (green), AGGF1 (red) and DAPI in ischemic gastrocnemius muscle of CTR and Gsα^ECKO^ mice. Scale bar, 20 μm. *n* = 3, **P* < 0.05 vs. CTR (unpaired Student’s *t*-test). **(C)** Immunofluorescent staining of CD31 (green), AGGF1 (red) and DAPI in gastrocnemius muscle of mice infected with LacZ or AGGF1 adenovirus. Scale bar, 20 μm. *n* = 3, ^**^*P* < 0.01 vs. LacZ (unpaired Student’s *t*-test). Data are mean ± SEM.
