# Supplementary figures and images for: Endothelial G protein stimulatory α-subunit is a critical regulator of post-ischemic angiogenesis
Source: Front Cardiovasc Med. 2022 Jul 25;9:941946. doi: 10.3389/fcvm.2022.941946 (PMC9358140; doi:10.3389/fcvm.2022.941946)

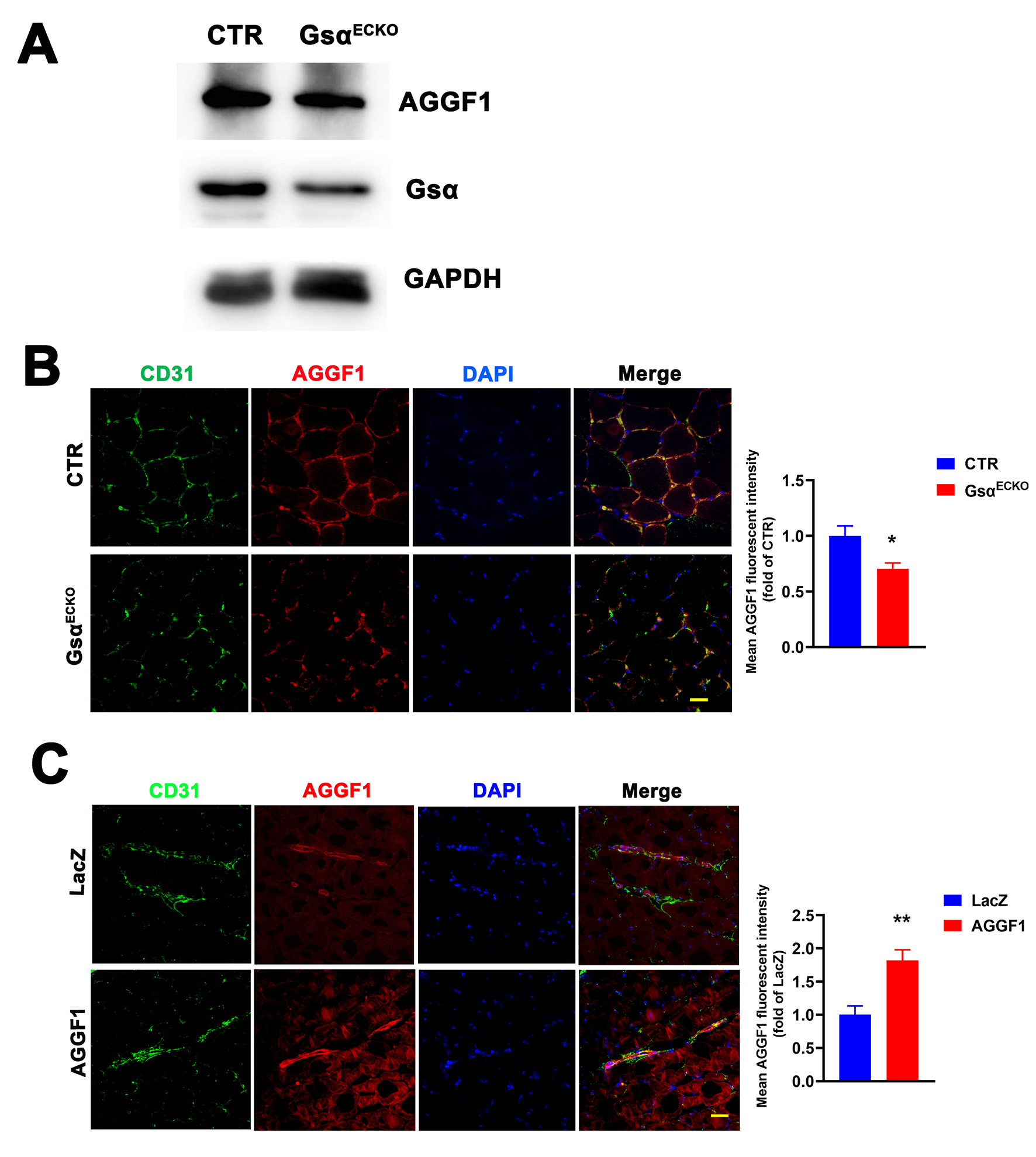

Supplement: Supplementary Figure 1 — Deletion of Gsα in endothelial cells reduced the expression of AGGF1. (A) Western blot of AGGF1 in isolated lung endothelial cells from CTR and GsαECKO mice. (B) Immunofluorescent staining of CD31 (green), AGGF1 (red) and DAPI in ischemic gastrocnemius muscle of CTR and GsαECKO mice. Scale bar, 20 μm. n = 3, *P < 0.05 vs. CTR (unpaired Student’s t-test). (C) Immunofluorescent staining of CD31 (green), AGGF1 (red) and DAPI in gastrocnemius muscle of mice infected with LacZ or AGGF1 adenovirus. Scale bar, 20 μm. n = 3, **P < 0.01 vs. LacZ (unpaired Student’s t-test). Data are mean ± SEM. [file Image_1.TIF]
